# Development of a rapid multiplex SSR genotyping method to study populations of the fungal plant pathogen *Zymoseptoria tritici*

**DOI:** 10.1186/1756-0500-7-373

**Published:** 2014-06-18

**Authors:** Angélique Gautier, Thierry C Marcel, Johann Confais, Charles Crane, Gert Kema, Frédéric Suffert, Anne-Sophie Walker

**Affiliations:** 1 UR 1290 BIOGER-CPP, INRA, BP01, Avenue Lucien Brétignières, F-78850 Thiverval-Grignon, France; 2 USDA-ARS, Crop Production and Pest Control Research Unit, Department of Botany and Plant Pathology, Purdue University, 915 West State Street, West Lafayette, IN 47907-2054, USA; 3 Plant Research International, Biointeractions and Plant Health, P.O. Box 16, 6700 AA Wageningen, The Netherlands

**Keywords:** Microsatellite, Single sequence repeat (SSR), Genotyping, Multiplex, Population genetics, Diversity, *Mycosphaerella graminicola*, Phytopathogenic fungus

## Abstract

**Background:**

*Zymoseptoria tritici* is a hemibiotrophic ascomycete fungus causing leaf blotch of wheat that often decreases yield severely. Populations of the fungus are known to be highly diverse and poorly differentiated from each other. However, a genotyping tool is needed to address further questions in large collections of isolates, regarding regional population structure, adaptation to anthropogenic selective pressures, and dynamics of the recently discovered accessory chromosomes. This procedure is limited by costly and time-consuming simplex PCR genotyping. Recent development of genomic approaches and of larger sets of SSRs enabled the optimization of microsatellite multiplexing.

**Findings:**

We report here a reliable protocol to amplify 24 SSRs organized in three multiplex panels, and covering all *Z. tritici* chromosomes. We also propose an automatic allele assignment procedure, which allows scoring alleles in a repeatable manner across studies and laboratories. All together, these tools enabled us to characterize local and worldwide populations and to calculate diversity indexes consistent with results reported in the literature.

**Conclusion:**

This easy-to-use, accurate, repeatable, economical, and faster technical strategy can provide useful genetic information for evolutionary inferences concerning *Z. tritici* populations. Moreover, it will facilitate the comparison of studies from different scientific groups.

## Background

*Zymoseptoria tritici* (syn. *Mycosphaerella graminicola,* anamorph *Septoria tritici*) [1] is an ascomycete fungus that causes Septoria leaf blotch, the most important foliar disease of winter wheat in Northern Europe. Moreover, this species has adapted to fungicides (especially from the azole and strobilurin families), which have been used intensively since the 1970s [2,3]. Intensive research has been undertaken to understand the biology of this fungus and the mechanisms of its pathogenicity, with the objective to develop durable management tools [4]. The *Z. tritici* genome has been fully sequenced and annotated, which revealed a high chromosome plasticity [5]. The sequenced genome contained 21 chromosomes, eight of them being facultatively gained or lost during meiosis in some isolates and constituting the dispensome, which accounts for up to approximately 12% of the genome size [5,6]. It was recently demonstrated that these accessory chromosomes originated mainly from ancient core chromosomes through a degeneration process that included breakage-fusion-bridge cycles, nondisjunction and mutational decay of duplicated sequences [7].

*Z. tritici* populations have been shown to be highly diverse on scales going from leaf to continents [8]. Wholesale gene flow and frequent recombination are supposed to occur at a global level [9]. Nevertheless, population differentiation has been detected in Iran, western USA, Germany and France [9-13], *i.e.* several of these areas were not included in the previous worldwide surveys. Additional large collections of isolates need to be analyzed to confirm and refine the population differentiation observed in those specific areas, and to identify the factors that influence the differentiation (for example, anthropogenic selection pressure or host/cultivar adaptation; seasonal adaptation; history of geographic spreads). Those questions should be answered in parallel on the so-called core genome (chromosomes 1 to 13) and the accessory (or dispensable) genome (chromosomes 14 to 21), which are characterized by strikingly different organizations [6,7] and rates of evolution [14] likely to result in diverging dynamics between the two compartments. Such a compartmentalized genome has been termed a “2-speed genome” [7].

Several marker sets are available to study the genetic structure of *Z. tritici* populations, ranging from sequence of mating-type genes [15-17], random amplified polymorphic DNA (RAPD) [18], restriction fragment length polymorphism (RFLP) [8] to amplified fragment length polymorphism (AFLP) [11,19]. A limited set of microsatellite/simple sequence repeat (SSR) markers was developed in the late 1990s [20] and used for field studies [10,21]. It was later complemented by a larger set of 99 new SSRs, derived from an EST database [22], and used at least in one study [23]. SSRs are of particular value for population studies, since they are co-dominant, easy to use, and highly polymorphic markers. As diversity in *Z. tritici* is unanimously considered high, a reduced panel of adapted SSRs should suffice to analyze population genetic structure [17]. Ideally, such markers should be genetically independent, *i.e.*, reside on different chromosomes, and selectively neutral to avoid bias in subsequent population genetic analyses [24]. Moreover, multiplex PCR, *i.e.*, the simultaneous amplification of several markers within a single reaction, is a good way to improve the SSR genotyping efficiency and to considerably reduce cost and labour [25].

Here, we optimize and complete a method that enables the amplification of 24 highly informative SSRs from *Z. tritici*, multiplexed into three independent panels.

## Findings

### Development of three efficient and multiplexed panels of SSRs for population studies

**Figure 1:**
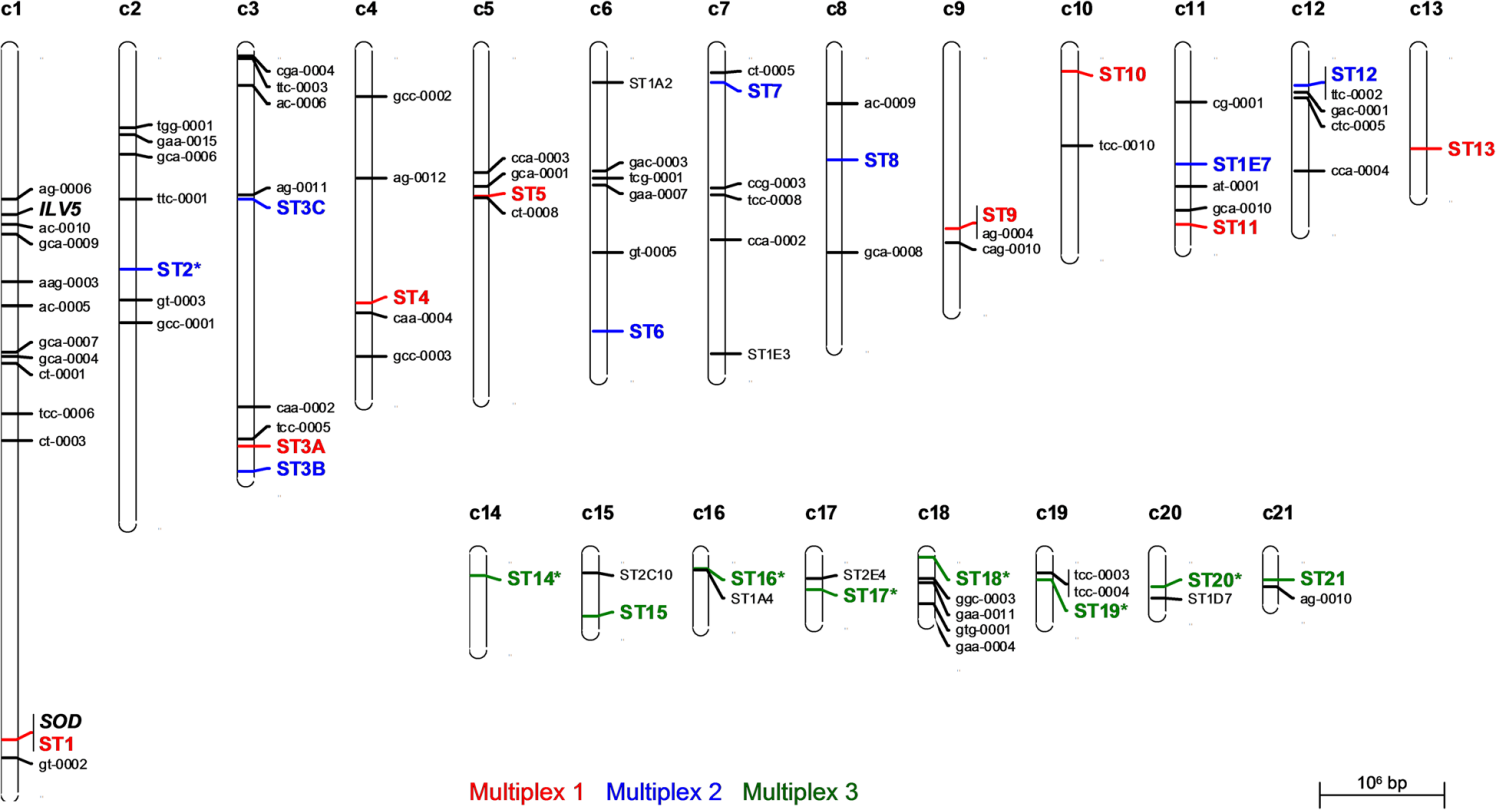
**Position of 80 published microsatellite loci on the genome of *Zymoseptoria tritici *(scale of the chromosomes is in base pairs) **[20,22]**.** The graphical representation of the map was constructed using the software MapChart [33]. The microsatellites used in the three multiplexes are represented in larger font and in bold with their new name (correspondence to the previously published name can be found in Table 1). The colour of a microsatellite locus indicates in which multiplex the marker is integrated: red for multiplex 1, blue for multiplex 2 and green for multiplex 3. An asterisk indicates that the marker is reported for the first time. The positions of the housekeeping genes *ILV5* (ID 102127) and *SOD* (ID 102956) are also indicated on chromosome 1.

To ensure relative independence of markers, and to detect the variably present accessory chromosomes, at least one SSR was chosen per chromosome. Therefore, the 9 [20] and 99 [22] previously developed SSRs were mapped *in silico* by blast-searching their primers against the *Z. tritici* genome at the JGI Genome Portal website (http://genome.jgi-psf.org/) [5]. The chromosome position of 7 and 73 SSRs was finally determined in each marker set, respectively (Figure 1). Blast of the primers from the remaining markers yielded non-specific or no result (Table 1). Interestingly, the position of 21 from the 23 SSRs genetically mapped by Goodwin *et al.*[22] was confirmed while the last 2 could not be positioned *in silico*.

**Table 1:** **Composition of three microsatellite PCR multiplexes developed for *Zymoseptoria tritici *** **population genetic studies**

**Multiplex**^**a**^	**Chrom.**	**Locus**^**b**^	**Locus in literature**^**c**^	**Repeat motif of IPO-323**^**d**^	**Primer sequences (5'- > 3')**	**Fluores-cent dye**^**e**^	**Size range (bp)**	**Protein ID**^**f**^	**Na**^**g**^	**Ne**^**h**^	**Fna**^**i**^	**Gene diversity (*H***_***E***_**)**^**j**^	
									***Total pop. (n = 166)***	***Total pop. (n = 166)***	***Total pop. (n = 166)***	***Local pop. (n = 33)***	***Worldwide pop. (n = 133)***
**Multiplex 1 (core chromosomes)**	1	ST1	GAC0002	(GAC)_6_	F: AATCGACCCCTTCCTTCAAC	Black	192-222	33894	3	1.4	0.6%	0.305	0.308
					R: GGGGGAGAGGCATAGTCTTG								
	3	ST3A	AG0003	(AG)_15_	F: ACTTGGGGAGGTGTTGTGAG	Black	226-258	103950	16	5.8	2.4%	0.785	0.825
					R: ACGAATTGTTCATTCCAGCG								
	4	ST4	AC0002	(AC)_7_	F: TGAACATCAACCTCACACGC	Green	182-206	71387	7	2.0	0.0%	0.479	0.504
					R: AGAAGAGGACGACCCACGAG								
	5	ST5	GGC0001	(GGC)_8_	F: GATACCAAGGTGGCCAAGG	Green	232-256	104484	6	1.4	0.0%	0.114	0.345
					R: CACGTTGGGAGTGTCGAAG								
	9	ST9	CT0004	(CT)_6_	F: CACCTCACTCCTCAATTCCG	Green	336-348	62567	6	1.7	3.0%	0.404	0.413
					R: GAAAGGTTGGTGTCGTGTCC								
	10	ST10	CAA0003	(CAA)_7_	F: TCCGTCATCAACAACACCAG	Blue	139-160	62856	6	2.7	0%	0.624	0.631
					R: TGGCCGTAGAACTGCTGAG								
	13	ST13	AG0009	(AG)_10_ (GGCA)_3_	F: GACTCCATTTACCTGTGGCG	Blue	192-200	106529	5	2.4	0%	0.566	0.587
					R: TGTGAAGGACACGCAAAGAG								
	11	ST11	GCA0002	(GCA)_6_	F: GTGTGAAACGAAAAGCGGAG	Blue	293-296	-	2	1.1	17.5%	0.071	0.087
					R: TACTGGGTATCGAGATCGGC								
**Multiplex 2 (core chromosomes)**	8	ST8	CT0007	(CT)_6_	F: TGCAGGGCATTTAATTGAGG	Black	180-188	61457	5	1.6	40.4%	0.322	0.404
					R: TCCATCCATTTAGGCTCGTC								
	12	ST12	TCC0002	(TCC)_6_	F: GAATCCACCTCTTCCTTGCC	Black	226-232	111480	3	1.1	5.4%	0.059	0.137
					R: AGGAGGATATCAAGGCCCAG								
	3	ST3B	CAA0005	(CAA)_8_	F: AAGAATCCCACCACCCAAAC	Black	263-299	-	10	2.9	0.6%	0.663	0.648
					R: CACACGGCTCCTTTGACAC								
	6	ST6	TCC0009	(TCC)_8_	F: TCAATTGCCAATAATTCGGG	Green	161-179	73474	6	1.5	3.6%	0.242	0.342
					R: AGACGAGGCAGTTGGTTGAG								
	3	ST3C	GCA0003	(GCA)_7_	F: TCCTATCAACTCCCGAGACG	Green	229-253	108534	5	2.2	1.2%	0.580	0.533
					R: CCGCACGTAGGAATTTTCAG								
	2	ST2	Chr02-140	(GCT)_12_	F: ACACCAAAGAAGGATCCACG	Green	338-365	108062	9	2.9	0%	0.439	0.689
					R: GCCGGAGGTCTATCAGTTTG								
	11	ST1E7	ST1E7	(CGG)_3_	F: GATCTCGAGCAGGGCGGAAGT	Blue	86-98	-	4	1.5	20.0%	0.204	0.373
					R: TCACACGCTGGTCTGTGAATC								
	7	ST7	AC0001	(AC)_21_	F: CACCACACCGTCGTTCAAG	Blue	171-227	105049	15	1.9	0.6%	0.463	0.487
					R: CGTAAGTTGGTGGAGATGGG								
**Multiplex 3 (accessory chromosomes)**	15	ST15	AC0007	(AC)_15_	F: TGCTCGCAAGACATAAAACG	Black	92-152	106622	15	8.0	16.9%	0.858	0.885
					R: CTCTTAGCATTGGTCGGTGG								
	21	ST21	GGA0001	(GGA)_6_	F: GTACGACACGGGCTATGGAG	Black	149-161	111806	5	1.8	32.5%	0.632	0.640
					R: GGCGATGACGATGAAACC								
	18	ST18	Chr18-007^*^	(GGA)_5_	F: AGAGGCAATGGTGGGTGAT	Black	245-272	111768	2	1.2	71.1%	0.411	0.430
					R: TCCTCCTCGTCCGACAATAC								
	19	ST19	Chr19-031	(TCG)_6_	F: CTACTGTATTTCCCGGGGGT	Black	351-363	97944	5	2.3	12.6%	0.700	0.631
					R: ACCCCTCTCTCCTTCTCTCG								
	20	ST20	Chr20-066^*^	(GAA)_22_	F: CTCCTCCACATCCATCCAAC	Green	229-289	-	19	13.9	10.8%	0.891	0.914
					R: GACGGAGGGGGAGAGGTAT								
	16	ST16	Chr16-016	(GAA)_6_	F: TTCACGGTATCACAGACCCA	Green	496-538	97708	7	1.2	13.3%	0.369	0.364
					R: GGGTTGTCCAAGCTGTTGTT								
	17	ST17	Chr17-040^*^	(AGA)_5_	F: ACAAGAGGCGGAAGACTGAA	Blue	236-239	97821	2	1.0	18.7%	0,367	0.311
					R: TCATGCGTCGTATTCTTGGA								
	14	ST14	Chr14-023	(TCC)_20_ (TCG)_5_	F: CAAACAGCCAGATCCACCTT	Blue	361-529	111734	36	12.1	13.8%	0.898	0.920
					R: GTCGTGGTCGGAGAGAGAAG								

^a^Primers for the housekeeping gene SOD are added in multiplexes 1 and 3 (green fluorescent dye; 96 bp amplicon; F:CTACCGATGCTCCAGCTGAGA and R:CACCACCCTTGCCATCCA), and primers from the housekeeping gene ILV5 are added in multiplexes 1 and 2 (black fluorescent dye; 108 bp amplicon; F:GCTCTATCGCCATCTTCCAGG and R:TCCTTCTCGAAGGTGGTCTTGT).

^b^Proposed name of microsatellite loci starting with the fungus initials and followed by the chromosome number on which the microsatellite is located (except for ST1E7).

^c^Loci name found in the literature [20,22] or in the Additional file 1 (primers for Chr18-007, Chr20-066 and Chr17-040 have been modified from the original design to fit the multiplex range size requirements).

^d^Repeat motif of IPO-323 as published in [20,22], with exception of ST7 and ST13, for which the motif was found slightly different after sequencing.

^e^Primers were purchased from Sigma Aldrich (France). They are WellRed oligos, licensed by Beckman Coulter (USA), and bearing fluorochromes at their 5’ end: D1: red, D2: black, D3: green, D4: blue.

^f^Identity number of the protein corresponding to SSR loci identified within expressed sequences (Filtered Gene Models from Mgraminicolav2 at JGI).

^g^Number of alleles identified in the total population (excluding null alleles).

^h^Effective number of alleles (excluding null alleles).

^i^Frequency of missing data or null alleles (no amplification) in the total population.

^j^Unbiased gene diversity *H*_*E*_ calculated as the probability that two individuals taken at random have different genotypes (excluding null alleles) [31].

Additionally, in order to increase the number of available SSRs from which to choose markers to constitute the panels, microsatellites were mined from the fasta-formatted genome of strain IPO-323 using the same pipeline of Perl scripts and C++ program that has been used to obtain microsatellites from EST sequences of higher plants [26]. This pipeline output primer sequences, motifs and repeat counts of simple and compound microsatellites, primer annealing temperatures, and primer locations in the genome. It searched for motifs of one to four nucleotides, with respective minimum repeat counts of 12, 6, 4, and 3. Up to 10 percent of mismatching positions were allowed, and the maximum distance between consecutive repeat blocks was five nucleotides in compound microsatellites. Primers were chosen to amplify products 100 to 500 nucleotides long, and to anneal at 60 ± 1.2°C, with preference to annealing temperatures in the interval 60.0 ± 0.2°C. In addition to the assembled genome of IPO-323, 86,776,768 short (45-base) reads were available for strain IPO-94269 (unpublished data). Reads with sufficiently high quality scores were mapped to the genome of IPO-323 by blastn, and a Perl-collated consensus of these reads provided an approximate assembly of the genome of IPO-94269. IPO-94269 lacks the accessory chromosomes 18 and 20, present in IPO-323 [5]. Otherwise, it was assumed for the sake of tractability that the two strains differed only in substitutions, and not in indels. Another Perl script blast-aligned the microsatellite primers from above to the IPO-323 and IPO-94269 genomes, and selected the primer pairs that hit each genome exactly once. For chromosomes 18 and 20, we selected the primer pairs that hit the genome of IPO-323 exactly once. Together, these were the 3781 “specific” microsatellites that are expected to produce single products from both genomes. Details on these SSRs are available in Additional file 1. A subset of 119 microsatellites having at least 50, 17, 10, or 8 repeats respectively for motifs of 1, 2, 3, or 4 nucleotides is also available in Additional file 2.

Thereafter, markers were chosen according to (1) their chromosomal location, (2) their polymorphism, (3) their allele size range, (4) their specificity towards related species and (5) the annealing temperature of their primers. For those reasons, candidate markers were selected in priority from the published sets because their polymorphism and species specificity had previously been assessed in a number of isolates [16,21,23]. SSR markers from core chromosomes were assembled in multiplexes 1 and 2 (8 markers per multiplex), and SSR markers from accessory chromosomes were combined in multiplex 3. The forward primers were labeled with fluorescent dyes (blue, green or black from Sigma-Aldrich®) in a way that the same color was given only to markers with non-overlapping range of allele sizes. Candidate markers were individually tested on eight representative *Z. tritici* DNAs from diverse origins, and on DNAs from fungal species closely related to *Z. tritici* and of agronomical importance [27], including three *Zymoseptoria passerinii*, one *Stagonospora nodorum* and one *Mycosphaerella fijiensis.* When markers were clearly amplified, were specific to *Z. tritici* and were found polymorphic between isolates, they were assembled in multiplexes and tested on the same DNA samples. According to the band patterns observed on high resolution agarose gel, multiplexes were optimized by means of marker exchange until all markers within a multiplex produced the expected amplification pattern at the consensus annealing temperature of 57°C, with the exception of multiplex 3 for which null alleles were allowed. The marker ST1E7 [20] was included in multiplex 2 to allow comparison with previously published datasets [10]. Finally, primers amplifying a 108 bp fragment from the housekeeping gene ILV5, keto-acid reductoisomerase (Chromosome 1, Protein ID 102127), and a 96 bp fragment from the housekeeping gene SOD, superoxide dismutase (Chromosome 1, Protein ID 102956), were introduced in the multiplexes to serve as amplification controls. This is particularly important for multiplex 3, since isolates without any accessory chromosome may be detected in field populations.

Once the three panels were optimized on the limited number of samples, the procedure was automatized for high-throughput routine work. Fresh sporidia, collected from 5 day-old mono-pycnidia cultures on malt-yeast agar MYA (20 g.l^−1^ malt extract, 5 g.l^−1^ yeast extract and 12.5 g.l^−1^ agar), were distributed in 96-well plates of the following extraction kit and ground for 90 s at 50 rps with a tungsten bead in 50 μl lysis (AP-1) buffer (65°C) with a Retsch-MM300 grinder. DNA extraction was carried-out using the Qiagen DNA ‘Biorobot 3000’ extraction robot and the Qiagen DNeasy Plant Maxi Kit, following the manufacturer’s protocol. After extraction, DNAs were quantified on a Nanodrop spectrophotometer (Thermo Scientific®), adjusted to 15 ng.μl^−1^ and stored at −20°C until use. 10 μl PCR reactions were performed using the Type-it microsatellite kit (Qiagen), specifically designed for multiplexed PCRs. Each reaction contained 2 μl deionized water, 1 μl Q-solution, 1 μl of the primer mix (containing 2 μM of each SSR/housekeeping gene primers), 5 μl of the Type-it mix and 1 μl DNA. Panel composition is detailed in Table 1. PCR conditions were identical for all multiplexes, with preheating at 95°C for 5 mn, followed by 30 cycles of 95°C for 30 s, 57°C for 90 s and 72°C for 30 s, with a final extension step of 60°C for 30 mn, using a MJ research PTC-200 thermocycler. The PCR products were run on a 2% agarose gel to check for amplification products. Sequencing reactions were prepared as follows: for multiplexes 1 and 2, 0.8 μl PCR product, 40 μl Sample Loading Solution (SLS) and 0.5 μl 400 bp size standard (red dye); the mix for multiplex 3 was the same, with the exception that 0.5 μl 600 bp size standard was used instead of the previous one. Fragments were separated using a Beckman Coulter CEQ-8000 DNA Analyzer. Results were visualized using the CEQ-8000 Genetic Analysis System Software (Beckman Coulter).

### Automation of allele scoring

We automated the allele scoring procedure using the binning analysis function of the CEQ-8000 Genetic Analysis System Software (Beckman Coulter) on the 166 genotyped samples (see below for description of populations). Such automation helps on one hand to solve problems related to the size assignment of ambiguous peaks produced on the chromatogram by Taq polymerase stuttering or fluorescent dyes reflection, and provides on the other hand a reproducible scoring method across readers and laboratories. In short, the software first provides for each marker a distribution of peak height and approximate size relative to the size-standard ladder. Each marker was then manually delimited (‘binned’) for each allele according to its size variability and a minimal peak height, in order to eliminate false positive or aberrant peaks and to minimize bin width. Binned alleles were finally assigned a definitive name (generally derived from the mean allele size) and allele size ranges were established (Table 1). The generated binning files could be used to annotate allele peaks from new data in any laboratory using a Beckman Coulter DNA Analyzer, and are described in Additional file 3 and provided as Additional file 4 or upon request to the authors. The alleles’ sizes of IPO-323 and other 100 reference isolates are provided as examples of this procedure (Additional file 3) to help further assignment by other operators. Resequencing data of all these reference isolates will be soon released on the JGI Genome Portal website (http://genome.jgi-psf.org/). This procedure assures reliable and accurate allele scoring, even when different or inexperienced operators manipulate the data, increasing the reproducibility between distinct datasets generated over time. This procedure can also be progressively improved, since new alleles of the same markers and also completely new markers can be implemented in the “binning files”.

### Checking the quality of the multiplexed SSR panels

Frequency parameters were calculated for the 166 individuals (see below for the description of the populations) with the software GenAlEx 6.4 [28]. For each marker, the number of SSR repeats present in the isolate IPO-323 was determined according to its genome sequence and used to infer the number of repeats from all the other alleles. In a few cases, fewer than 0 repeats were deduced for alleles present in never more than one to three individuals; such alleles were coded as missing alleles and were not considered further. Excluding those, a total of 204 alleles were detected. The number of alleles per SSR ranged from 2 to 16 for panels 1 and 2 on the core chromosomes (Table 1). Markers on the accessory chromosomes 14 and 20 had particularly numerous alleles with 36 and 20 detected alleles, respectively. The 3 markers ST11 in panel 1, and ST8 and ST1E7 in panel 2 could not be amplified in a large number of isolates probably because of the presence of null alleles (Table 1). The same observation is true for all markers from panel 3 on the accessory chromosomes with 11 to 71% null amplifications, on chromosomes 20 and 18, respectively. This result is supported by other studies, which revealed that chromosome 18 is the accessory chromosome the most often missing in field isolates of *Z. tritici*[7]. We also sequenced the PCR product of ST18 from 3 isolates carrying the 245 bp allele (IPO-02166, IPO-98032, IPO-95054) and 2 isolates carrying the 272 bp allele (IPO-98038, IPO-95052) (Table 1) and found that this marker was not polymorphic at its microsatellite motif (GGA)_5_. The allele size difference at ST18 is caused by an insertion of 27 bp in the larger allele.

The eight accessory chromosomes exhibit strong differentiation between strains, in number but also in length of chromosomes [7]. Indeed, the frequency in presence or absence of chromosomal segments varies strongly along each accessory chromosome. For example, chromosome 14 is present in more than 80% of the strains while it harbors a substantial insertion present in less than 20% of these same strains [7]. Except for chromosome 18, the SSR markers from panel 3 on accessory chromosomes are in conserved segments of their respective chromosome, which make them reliable indicators of the presence or absence of each accessory chromosome. The presence or absence of chromosome 18 is particularly difficult to predict due to its high differentiation between strains from different geographical origins [7].

**Figure 2:**
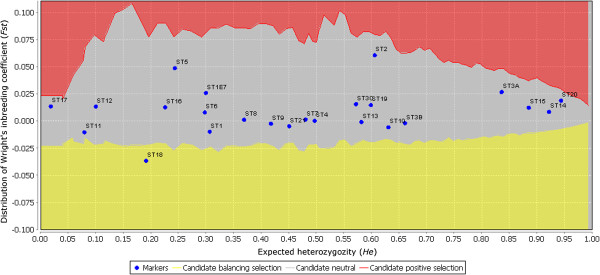
**Detection of outlier SSR loci using the Lositan workbench at the 95% threshold.** In red, the confidence area for candidate loci under positive selection; in grey, the confidence area for neutral loci; in yellow, the confidence area for candidate loci potentially under balancing selection. The names of the loci are indicated close to their position on the chart.

Non-neutral loci may bias population genetic studies [24]. To identify possible outlier loci in the panels, the 24 SSR markers constituting the three multiplex panels were subjected to analysis with the selection detection workbench Lositan[29]. Lositan detects outlier loci from the expected distribution of Wright’s inbreeding coefficient *F*_*st*_ versus the expected heterozygozity (or gene diversity) *H*_*e*_ under an island model of migration with neutral markers. A neutral mean *F*_*st*_ was computed after excluding loci for which selection had been detected initially. The mean observed *F*_*st*_ value was 0.005, while individual marker *F*_*st*_ values ranged from −0.036 to 0.061. Only one out of the 24 SSR markers, ST18, was declared an outlier, since it appeared in the confidence area for loci potentially under balancing selection (Figure 2). Either directional selection or genotyping errors can generate outlier effects. The very high proportion (71%) of null alleles for ST18 is the most likely explanation for this marker being detected as an outlier. The other markers are considered as neutral.

### Validation of the multiplexed SSR panels: diversity measurement in two populations

To validate the SSR multiplexing procedure, the automated allele assignment, and the value of the selected SSR markers for population genetic studies, two distinct fungal populations were genotyped following the proposed protocol. Isolates belonging to a first, global, population (n = 133) were collected worldwide over 25 years; they covered 28 countries from the five continents, including different areas in France, and were retrieved from laboratory collections. Isolates belonging to a second, local, population (n = 33) were collected during the same crop season 2009–2010 in a single experimental field plot in Grignon, France (48°51'N, 1°58'E; [30]). Samples were genotyped and analyzed as previously described.

**Figure 3:**
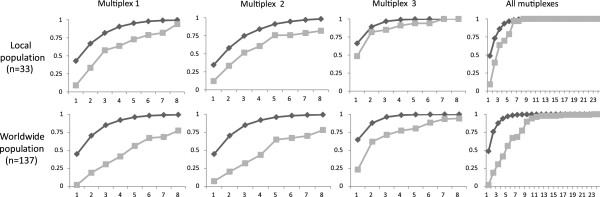
**Clonal richness and gene diversity measured in local and worldwide *Zymoseptoria tritici *populations according to the number of SSRs used in the analysis**^**a**^**. **^a^Clonal richness, measured as the number of unique genotypes compared to the number of isolates (G/N) is represented with grey squares. Gene diversity, estimated as the mean expected heterozygosity over loci [31] is represented with black diamonds. Both indexes are calculated for each multiplex and for all of them according to the number of SSR markers involved in the calculation.

For further index calculations, missing data were coded as missing alleles for multiplexes 1 and 2, and as null alleles for multiplex 3. Gene diversity, estimated as the mean expected heterozygoty *H*_*e*_ over the loci [31], and clonal richness (G/N; number of unique genotypes compared to the number of individuals) were plotted against the number of loci used to calculate these indexes in the three multiplexes and for all of them (Figure 3), using the Multilocus program [32]. As expected, gene diversity was similarly high in the two populations, and exceeded 0.95 after 4 loci were included in calculation for multiplexes 1 and 2, and after two loci were included for multiplex 3, probably because null alleles are considered as distinct alleles for markers located on the dispensome. The 24 SSR loci present in the three panels are then sufficient to saturate the whole gene diversity, at a local and a larger scale. The observed ranges for gene diversity, for single loci (Table 1) or for genotypes (Figure 3), are similar or even greater compared to what has been reported in the literature for various *Z. tritici* populations, either with SSR, RAPD, AFLP or RFLP markers [9-13,18]. The 24 SSR loci are also sufficient to detect possible clonal individuals as only 11 and 23 markers were necessary to distinguish all haplotypes in the local and world populations, respectively (Figure 3); in practice 164 haplotypes were recognized among the 166 isolates. Such a low proportion of clonal individuals (G/N) corroborates several studies pointing out the importance of sexual recombination in field populations of *Z. tritici*[9,13,17,18,21].

## Conclusion

The proposed multiplex PCR genotyping method, together with the automatic allele assignment procedure, constitutes a reliable tool for population genetics studies on *Z. tritici*. Moreover, these ready-to-use methods enable important savings in terms of money, time, reproducibility and accuracy, and produce results in agreement with others previously published about *Z. tritici* populations. As the chosen SSRs are highly polymorphic and describe most of the population diversity, the three multiplex panels could be used “à la carte” to address specific questions. For example, the use of a single panel (either multiplex 1 or 2) could suffice to describe diversity in local/reduced populations, whereas both panels representing the core chromosomes (multiplexes 1 and 2 together) would be useful to study larger unknown populations. Multiplex 3 is of great interest to acquire complementary information on the dispensome dynamics and evolution [7] of the studied populations, but users should keep in mind that false negative results are always possible due to the variation in chromosomal content between strains. Multiplex 3 should also be used with care for population genetics analyses, as the great number of missing alleles may bias the results. The three markers with null alleles from the multiplexes 1 and 2 (*i.e.* ST11, ST8, ST1E7) may be as well omitted from certain analyses for the same reason. These sets of optimized markers will be of great relevance to the *Z. tritici* scientific community, as they provide a method that can be applied in any laboratory and will allow facile comparison of studies from various locations, scales, and with different aims. The method is currently being used successfully in two laboratories to genotype different European populations consisting of several thousand individuals.

## Competing interest

The authors declare that they have no competing interest.

## Authors’ contributions

AG designed and optimized the multiplexes. JC genotyped the populations. TM localized the SSRs on the genome, designed primers and selected candidate markers. CC identified the new SSRs from the genome sequence. GK and FS provided the worldwide and local populations, respectively. ASW led the project and developed allele automatic assignment. ASW and TM analyzed the data and wrote the manuscript. All authors read and approved the final manuscript.

## Supplementary Material

Additional file 1 **Characteristics of 3781 “specific” microsatellites identified *in silico *** **from *Zymoseptoria tritici *** **genome.** Primer pairs are proposed for each microsatellite, which hit each genome of strains IPO-323 and IPO-94269 exactly once. Click here for file

Additional file 2 Subset of 119 microsatellites with highly repeated motifs. Click here for file

Additional file 3 **Alleles computed in the automated allele scoring procedure and frequency of alleles recorded among 166 individual strains.** Strains were selected from a local French population and from a worldwide collection.The multilocus genotypes of 101 strains is provided as reference. Click here for file

Additional file 4 **Binning files, to be used for automatic allele scoring.** Files to use with the CEQ-8000 Genetic Analysis System Software (Beckman Coulter) to provide automatic assignment of alleles for each SSR marker. Click here for file

## References

[B1] Quaedvlieg W Kema GHJ Groenewald JZ Verkley GJM Seifbarghi S Razavi M Gohari AM Mehrabi R Crous PW *Zymoseptoria* gen. nov.: a new genus to accommodate *Septoria*-like species occurring on graminicolous hosts Persoonia 2011 26 57 69 10.3767/003158511X571841 22025804 PMC3160802

[B2] Stammler G Carstensen M Koch A Semar M Strobel D Schlehuber S Frequency of different CYP51-haplotypes of *Mycosphaerella graminicola* and their impact on epoxiconazole-sensitivity and -field efficacy Crop Prot 2008 27 1448 1456 10.1016/j.cropro.2008.07.007

[B3] Leroux P Walker A Multiple mechanisms account for resistance to sterol 14α-demethylation inhibitors in field isolates of *Mycosphaerella graminicola* Pest Manag Sci 2011 67 44 59 10.1002/ps.2028 20949586

[B4] Orton ES Deller S Brown JKM *Mycosphaerella graminicola*: from genomics to disease control Mol Plant Pathol 2011 12 413 424 10.1111/j.1364-3703.2010.00688.x 21535348 PMC6640266

[B5] Goodwin SB Ben M'Barek S Dhillon B Wittenberg AHJ Crane CF Hane JK Foster AJ Van der Lee TAJ Grimwood J Aerts A Antoniw J Bailey A Bluhm B Bowler J Bristow J van der Burgt A Canto-Canche´ B Churchill ACL Conde-Ferra`ez L Cools HJ Coutinho PM Csukai M Dehal P De Wit P Donzelli B van de Geest HC van Ham RCHJ Hammond-Kosack KE Henrissat B Kilian A Finished genome of the fungal wheat pathogen *Mycosphaerella graminicola* reveals dispensome structure, chromosome plasticity, and stealth pathogenesis Plos Genet 2011 7 6 e1002070 10.1371/journal.pgen.1002070 21695235 PMC3111534

[B6] Wittenberg AHJ van der Lee TAJ Ben M'Barek S Ware SB Goodwin SB Kilian A Visser RGF Kema GHJ Schouten HJ Meiosis drives extraordinary genome plasticity in the haploid fungal plant pathogen *Mycosphaerella graminicola* PLoS One 2009 4 6 e5863 10.1371/journal.pone.0005863 19516898 PMC2689623

[B7] Croll D Zala M McDonald BA Breakage-fusion-bridge cycles and large insertions contribute to the rapid evolution of accessory chromosomes in a fungal pathogen Plos Genet 2013 9 6 e1003567 10.1371/journal.pgen.1003567 23785303 PMC3681731

[B8] Linde CC Zhan J McDonald BA Population structure of *Mycosphaerella graminicola*: from lesions to continents Phytopathology 2002 92 946 955 10.1094/PHYTO.2002.92.9.946 18944019

[B9] Zhan J Pettway RE McDonald BA The global genetic structure of the wheat pathogen *Mycosphaerella graminicola* is characterized by high nuclear diversity, low mitochondrial diversity, regular recombination, and gene flow Fungal Genet Biol 2003 38 286 297 10.1016/S1087-1845(02)00538-8 12684018

[B10] El Chartouni L Tisserant B Siah A Duyme F Leducq JB Deweer C Fichter-Roisin C Sanssene J Durand R Halama P Reignault P Genetic diversity and population structure in French populations of *Mycosphaerella graminicola* Mycologia 2011 103 764 774 10.3852/10-184 21289103

[B11] Kabbage M Leslie JF Hulbert SH Bockus WW Comparison of natural populations of *Mycosphaerella graminicola* from single fields in Kansas and California Physiol Mol Plant Pathol 2009 74 55 59 10.1016/j.pmpp.2009.09.002

[B12] Abrinbana M Mozafari J Shams-bakhsh M Mehrabi R Genetic structure of *Mycosphaerella graminicola* populations in Iran Plant Pathol 2010 59 829 838 10.1111/j.1365-3059.2010.02309.x

[B13] Schnieder F Koch G Jung C Verreet JA Genotypic diversity of the wheat leaf blotch pathogen *Mycosphaerella graminicola* (anamorph) *Septoria tritici* in Germany Eur J Plant Pathol 2001 107 285 290 10.1023/A:1011256504146

[B14] Stukenbrock EH Jorgensen FG Zala M Hansen TT McDonald BA Schierup MH Whole-genome and chromosome evolution associated with host adaptation and speciation of the wheat pathogen *Mycosphaerella graminicola* Plos Genet 2010 6 12 e1001189 10.1371/journal.pgen.1001189 21203495 PMC3009667

[B15] Waalwijk C Mendes O Verstappen ECP de Waard MA Kema GHJ Isolation and characterization of the mating-type idiomorphs from the wheat septoria leaf blotch fungus *Mycosphaerella graminicola* Fungal Genet Biol 2002 35 277 286 10.1006/fgbi.2001.1322 11929216

[B16] Zhan J Kema GHJ Waalwijk C McDonald BA Distribution of mating type alleles in the wheat pathogen *Mycosphaerella graminicola* over spatial scales from lesions to continents Fungal Genet Biol 2002 36 128 136 10.1016/S1087-1845(02)00013-0 12081466

[B17] Gurung S Goodwin SB Kabbage M Bockus WW Adhikari TB Genetic differentiation at microsatellite loci among populations of *Mycosphaerella graminicola* from California, Indiana, Kansas, and North Dakota Phytopathology 2011 101 1251 1259 10.1094/PHYTO-08-10-0212 21692645

[B18] Razavi M Hughes GR Molecular variability of *Mycosphaerella graminicola* as detected by RAPD markers J Phytopathol 2004 152 543 548 10.1111/j.1439-0434.2004.00893.x

[B19] Schnieder F Koch G Jung C Verreet JA The application of molecular markers for genetic characterization of *Septoria tritici* populations Z Pflanzenk Pflanzens-J Plant Dis Prot 1998 105 452 461

[B20] Owen PG Pei M Karp A Royle DJ Edwards KJ Isolation and characterization of microsatellite loci in the wheat pathogen *Mycosphaerella graminicola* Mol Ecol 1998 7 1611 1612 9819910 10.1046/j.1365-294x.1998.00499.x

[B21] Razavi M Hughes GR Microsatellite markers provide evidence for sexual reproduction of *Mycosphaerella graminicola* in Saskatchewan Genome 2004 47 789 794 10.1139/g04-036 15499393

[B22] Goodwin SB van der Lee TAJ Cavaletto JR Hekkert BTL Crane CF Kema GHJ Identification and genetic mapping of highly polymorphic microsatellite loci from an EST database of the septoria tritici blotch pathogen *Mycosphaerella graminicola* Fungal Genet Biol 2007 44 398 414 10.1016/j.fgb.2006.09.004 17074520

[B23] Boukef S McDonald BA Yahyaoui A Rezgui S Brunner PC Frequency of mutations associated with fungicide resistance and population structure of *Mycosphaerella graminicola* in Tunisia Eur J Plant Pathol 2012 132 111 122 10.1007/s10658-011-9853-8

[B24] Negrini R D'Andrea M Crepaldi P Colli L Nicoloso L Guastella AM Sechi T Bordonaro S Ajmone-Marsan P Pilla F Econogene C Effect of microsatellite outliers on the genetic structure of eight Italian goat breeds Small Rumin Res 2012 103 99 107 10.1016/j.smallrumres.2011.08.006

[B25] Hayden MJ Nguyen TM Waterman A Chalmers KJ Multiplex-ready PCR: a new method for multiplexed SSR and SNP genotyping BMC Genomics 2008 9 80 10.1186/1471-2164-9-80 18282271 PMC2275739

[B26] Crane C Patterned sequence in the transcriptome of vascular plants BMC Genomics 2007 8 173 10.1186/1471-2164-8-173 17573970 PMC1940011

[B27] Goodwin SB Zismann VL Phylogenetic analyses of the ITS region of ribosomal DNA reveal that *Septoria passerinii* from barley is closely related to the wheat pathogen *Mycosphaerella graminicola* Mycologia 2001 93 934 946 10.2307/3761758

[B28] Peakall R Smouse PE GENALEX 6: genetic analysis in Excel. Population genetic software for teaching and research Mol Ecol Notes 2006 6 288 295 10.1111/j.1471-8286.2005.01155.x PMC3463245 22820204

[B29] Antao T Lopes A Lopes RJ Beja-Pereira A Luikart G LOSITAN: a workbench to detect molecular adaptation based on a F(st)-outlier method BMC Bioinformatics 2008 9 323 10.1186/1471-2105-9-323 18662398 PMC2515854

[B30] Suffert F Sache I Relative importance of different types of inoculum to the establishment of *Mycosphaerella graminicola* in wheat crops in north-west Europe Plant Pathol 2011 60 878 889 10.1111/j.1365-3059.2011.02455.x

[B31] Nei M Estimation of average heterozygosity and genetic distance from a small number of individuals Nature 1978 89 583 590 10.1093/genetics/89.3.583 PMC1213855 17248844

[B32] Agapow PM Burt A Indices of multilocus linkage disequilibrium Mol Ecol Notes 2001 1 101 102 10.1046/j.1471-8278.2000.00014.x

[B33] Voorrips RE MapChart: software for the graphical presentation of linkage maps and QTLs J Hered 2002 93 77 78 10.1093/jhered/93.1.77 12011185

